# The Nurses' Home

**Published:** 1907-10-12

**Authors:** 


### Nursing Administration.

### THE NURSES' HOME.

The first essential to success in the conduct of a nurses' home, whether it be in connection with a district nurses' association, a nursing institute, or a hospital, is to place it under the charge of a competent housekeeper, who should also be a nurse, and the second is to establish a good system of accounts, by means of which the cost of the home and the cost of the individual nurse can be clearly ascertained. Bad housekeeping and tangled accounts are at the root of all the muddle and extravagance so often glaringly manifested in connection with houses run for the convenience of nurses. It seems to be imagined by some people that all that is required in the upkeep of a nurses' home is to order the dinner and pay the bills. Accordingly it is left to the over-worked superintendent, whose nights may often be broken, and whose days are a perpetual struggle to fit 65 minutes into every hour, to cope with domestic problems which are sufficient to fill one person's entire time. Hence servants become lazy and tyrannical, tradesmen get the impression that anything will do for the nurses, and general discomfort, sometimes real hardships, are the order of the day. An attempt is often made in such ramshackle households to substitute rigorous rules for the regular supervision which can alone produce an atmosphere of order. Penalties are imposed for every trifling act of forgetfulness, yet conspicuous irregularities pass unnoticed. The orderly and punctual are penalised while those who are entirely selfish and indifferent to the comfort of others contrive to secure their own way. There are many such establishments where the appointment of a housekeeper would be resisted on grounds of economy by a committee or by the superintendent herself. Yet it could easily be proved that to pay £40 a year to a competent woman able from her own experience to understand the special requirements of the nurses in residence, would be to save more than twice as much in money alone, and in respect of health and saving to time and temper the gain would be incalculable. Nurses in residence between cases, liable to be called out at any moment, do not constitute altogether an easy household. Their food should be light and easy of digestion, very nourishing, and varied as far as seasonal changes admit. Their rooms should be conspicuously airy, and since several occupants may sleep in one room in the course of a week, the most scrupulous attention to cleanliness and neatness is imperatively demanded. Often a nurse will return thoroughly exhausted from an arduous spell of work, when her only desire will be to sleep the clock round undisturbed. If the housekeeper is kind and clever she will know how to secure this life-giving quiet to those who need it, and place them quickly in a position to resume work again, instead of reducing them to the necessity for wandering restlessly about in a state of over fatigue, harassed by avoidable noises, and hampered by regulations about meals. It ought to be the business of one in authority to watch for the comfort of the nurses, and regard the engagements of each member of the household as part of the common concern, instead of as irritating individual affairs. The housekeeper, in fact, in such a household is appointed to stand between the nurses and the "narrow things of home," thus setting them free to concentrate their entire strength and attention on their nursing duties. It is inconceivable what such a housekeeper can do to maintain the staff in contentment and health. The difference between returning to a homelike atmosphere between cases, and getting true refreshment of spirit and body and returning to encounter the old, old discomforts and indifference, is scarcely to be appreciated by those who have never tried the hardship of being merely an unconsidered item in an ill-managed household. It will be urged that nurses "encroach," that they are easily "spoiled," and love to be always complaining. We believe, on the contrary, that they are invariably most grateful for efforts made to meet their special convenience, and that such housekeepers as we have tried to picture would find a rich store of volunteer assistance in busy moments among the staff whose interests they serve. They are not unknown, and as their value gets to be recognised their number will no doubt increase. The important thing is to understand that there is ample scope for talents in this direction, and that no nurses' home can be a real success without the housekeeper.

Hardly less necessary is it to dissever the expenses of the nurses' home absolutely from all other outgoings. It ought not to be reckoned in the ordinary accounts of the association, mixed up with percentages and office expenses. If the home be housed under the same roof as the offices the rent ought to be divided, and the same is true of other expenses. An estimate can easily be framed of the amount due to each section of the business for servants' wages, fuel, lighting, and general expenditure, and the nurses' home should have its own separate statement of accounts, and be able to show the precise cost entailed by its upkeep for the nurses. It is necessary, also, to keep a register of nights spent at the home by each member of the staff. A list containing the names of all the members of the staff should be ruled for the month or quarter, and in the spaces thus prepared a cross should be entered on the correct date against the name of each one who spends a night in the home. At the end of the year each nurse should be informed how many nights have been spent at home, and by this means much vague dissatisfaction would be abolished. A good system when nurses are paid a salary, instead of receiving a percentage, is to allow some additional bonus when the cases have been so continuous that only small advantage has been obtained from the home. The actual expense of the home ought to be reckoned in apportioning the salary, and this expense should be clearly understood by those for whom it is run. It is for want of any perception of the inevitable heavy expense of keeping up such establishments that nurses often talk wildly about being "sweated," even in institutes which can barely pay their way.

